# Supplementary material for: MetaRibo-Seq measures translation in microbiomes
Source: Nat Commun. 2020 Jun 29;11:3268. doi: 10.1038/s41467-020-17081-z (PMC7324362; doi:10.1038/s41467-020-17081-z)
Supplement: Supplementary file 10 — Supplementary Data 7 [file 41467_2020_17081_MOESM10_ESM.zip › File2/Confidence_High_Taxonomy/134756_out.krona.html]

Javascript must be enabled to view this page.

members
magnitude
magnitudeUnassigned
count
unassigned
taxon
rank

134756\_out

11

superkingdom
11
2

11
1239
phylum

11
186801
class

order
186802
11

11
541000
family

216851
11
genus

853

SRS016132\_contig\_number\_3532SRS016203\_contig\_number\_10576SRS021484\_contig\_number\_15028SRS077502\_contig\_number\_11902SRS097889\_contig\_number\_32958SRS098061\_contig\_number\_contig-100\_1417.91264SRS104084\_contig\_number\_13725SRS143372\_contig\_number\_7985SRS144537\_contig\_number\_37025SRS148721\_contig\_number\_17561
10
species

species
1

SRS014412\_contig\_number\_21194
2302956
